# Association between maternal khat use and other determinants and low birth weight in Halaba Zone, South Ethiopia: an unmatched case–control study

**DOI:** 10.3389/fped.2025.1416027

**Published:** 2025-02-25

**Authors:** Biruk Wogayehu, Tsegaye Demissie, Eskinder Wolka, Mekuriaw Alemayehu

**Affiliations:** ^1^ Department of Public Health, Arbaminch College of Health Sciences, Arbaminch, Ethiopia; ^2^ Department of Public Health, School of Public Health, College of Medicine and Health Sciences, Wolaita Sodo University, Sodo, Ethiopia; ^3^ Department of Epidemiology and Biostatistics, Institute of Public Health, College of Medicine and Health Sciences, University of Gondar, Gondar, Ethiopia

**Keywords:** low birth weight, khat, pregnant, determinants, Ethiopia

## Abstract

**Introduction:**

Early newborn mortality, morbidity, and long-term health outcomes are significantly predicted by birth weight. Many babies are born underweight in Ethiopia, but few case–control studies have previously examined the risk variables associated with khat consumption and low birth weight (LBW). Therefore, the aim of this study was to identify maternal khat use and maternal sociodemographic and obstetric risk factors associated with LBW in the Halaba Kulito General Hospital, southern Ethiopia.

**Methods:**

A hospital-based case–control study design was used on 334 neonates (111 cases and 223 controls) at Halaba General Hospital in Halaba Zone, southern Ethiopia, from 01 October 2023 to 27 February 2024. A consecutive sampling method was used to select both the cases and controls. Bi-variable and multivariable logistic regression models were fitted using Stata 14.0 to estimate the effect of maternal khat use and other factors on low birth weight. A *p*-value of <0.05 was considered a significant difference in low birth weight between the cases and controls.

**Results:**

We found that the mean age of the cases and controls at birth was 25.4 ± 4.57 years and 24.2 ± 3.96 weeks, respectively. Illiteracy [adjusted OR (AOR) = 3.7, 95%CI 1.34, 10.45], rural residence (AOR = 4.1, 95%CI 1.51, 11.35), gestational age <37weeks (AOR = 16.5, 95%CI 7.05, 38.55), maternal mid-upper arm circumference (MUAC) <23 cm (AOR = 4.7, 95%CI 1.89, 11.65), weight gain <12 kg (AOR = 4.8, 95%CI 1.22, 18.59), monthly khat use (AOR = 9.5, 95%CI 2.13, 41.98), weekly khat use (AOR = 11.1, 95%CI 3.69, 33.40), and daily khat use (AOR = 14.1, 95%CI 4.74, 42.03) were the determinant factors for delivering a newborn with low birth weight.

**Conclusion:**

The evidence from this study suggests that illiteracy, rural residence, gestational age <37weeks, maternal MUAC <23 cm, weight gain <12 kg, monthly khat use, weekly khat use, and daily khat use were independent predictors of low birth weight. Suggested strategies involve the early identification and management of identified modifiable variables. We recommend that stakeholders in khat control commit to providing health education and awareness, incorporating khat use among women in the khat control policy, and designing interventions for the cessation of khat use among women.

## Introduction

While for most women, pregnancy and childbirth are happy experiences, there are instances when they result in unfavorable birth outcomes (1). Unfavorable birth outcomes are complex and mostly consist of preterm birth, stillbirth, low birth weight (LBW), macrosomia, congenital anomalies, and infant/neonatal death (2).

**Table 1:** Sociodemographic characteristics of mothers who gave birth at Halaba Kulito General Hospital, South Ethiopia.

Variable	LBW frequency (%)	NBW frequency (%)	Total frequency (%)
Maternal age (year)			
≤19	4 (3.6)	16 (7.2)	20 (6.0)
20–25	66 (59.5)	142 (63.7)	208 (62.3)
≥26	41 (36.9)	65 (29.1)	106 (31.7)
Maternal education			
Cannot read and write	65 (58.5)	68 (30.5)	132 (39.5)
Primary (1–8)	32 (28.8)	87 (39.0)	119 (35.6)
Secondary (9–12) and above	15 (13.5)	68 (30.5)	83 (24.9)
Maternal occupation			
Housewife	70 (63.1)	148 (66.4)	218 (65.3)
Merchant	26 (23.4)	52 (22.3)	78 (23.3)
Employed	15 (13.5)	23 (10.3)	38 (11.4)
Residence			
Rural	63 (56.8)	30 (13.4)	93 (27.8)
Urban	48 (43.2)	193 (86.6)	241 (72.2)
Religion			
Orthodox	11 (9.9)	37 (16.6)	48 (14.4)
Muslim	76 (68.5)	137 (61.4)	213 (63.8)
Protestant	24 (21.6)	49 (22.0)	73 (21.9)

LBW, low birth weight; NBW, normal birth weight.

**Table 2:** Obstetric and health-related characteristics of mothers who gave birth at Halaba Kulito General Hospital, South Ethiopia.

Variable	LBW frequency (%)	NBW frequency (%)	Total frequency (%)
Gravidity			
Primigravida	26 (23.4)	63 (28.3)	89 (26.6)
Multigravida	64 (57.6)	135 (60.5)	199 (59.6)
Grand multigravida	21 (18.9)	25 (11.2)	46 (13.8)
Parity			
Nullipara	29 (26.1)	65 (29.1)	94 (28.1)
Primipara	32 (28.8)	54 (24.2)	86 (25.7)
Multipara	50 (45.0)	104 (46.6)	154 (46.1)
Birth interval in years			
≤2	29 (26.1)	42 (18.8)	71 (21.3)
>2	82 (73.9)	181 (81.2)	263 (78.7)
Gestational age			
<37weeks^a^	93 (83.8)	30 (13.5)	123 (36.8)
≥37weeks	18 (16.2)	193 (86.5)	211 (63.2)
Number of antenatal care (ANC) visits			
1 to 3 ANC visits	77 (69.4)	73 (32.7)	150 (44.9)
≥4 ANC visits	34 (30.6)	150 (67.3)	184 (55.1)
Mode of delivery			
Emergency cesarean section	24 (21.6)	20 (9.0)	44 (13.2)
Spontaneous vaginal delivery	87 (78.4)	203 (91.0)	290 (86.8)
History of abortion			
Yes	11 (9.9)	6 (2.7)	17 (5.1)
No	100 (90.1)	217 (97.3)	317 (94.9)
Pregnancy-induced hypertension			
Yes	4 (3.6)	2 (0.9)	6 (1.8)
No	107 (96.4)	221 (99.1)	328 (98.2)

LBW, low birth weight; NBW, normal birth weight.

^a^ Newborns born between 30 and 32 weeks.

**Table 3:** Nutritional and substance use characteristics of mothers who gave birth at Halaba Kulito General Hospital, South Ethiopia.

Variable	LBW frequency (%)	NBW frequency (%)	Total frequency (%)
Maternal mid-upper arm circumference (cm)			
<23	59 (53.2)	34 (15.2)	93 (27.8)
≥23	52 (46.8)	189 (84.8)	241 (72.2)
Weight gain during pregnancy			
<12 kg	103 (92.8)	178 (79.8)	281 (84.1)
≥12 kg	8 (7.2)	45 (20.2)	53 (15.9)
Iron and folate supplementation			
Yes	91 (82.0)	216 (96.9)	307 (91.9)
No	20 (18.0)	7 (3.1)	27 (8.1)
Have you ever chewed khat?			
Yes	77 (69.4)	52 (23.3)	129 (38.6)
No	34 (30.6)	171 (76.7)	205 (61.4)
How often do you chew khat?			
Never	34 (36.6)	171 (76.7)	205 (61.4)
Monthly	13 (11.7)	11 (4.9)	24 (7.2)
Weekly	24 (21.6)	25 (11.2)	49 (14.8)
Daily	40 (36.0)	16 (7.2)	56 (16.8)
Time spent in khat sessions			
0	34 (30.6)	171 (76.7)	205 (61.4)
2–5 h	46 (41.4)	29 (13.0)	75 (22.4)
≥6 h	31 (27.9)	23 (10.3)	54 (16.2)
Amount of khat chewed per khat session (bundle)			
Non-user	34 (30.6)	169 (75.8)	203 (60.8)
Half a bundle	26 (23.4)	25 (11.2)	51 (15.3)
One bundle	51 (45.9)	29 (13.0)	80 (23.9)
Have you ever drunk alcohol?			
Yes	7 (6.3)	5 (2.2)	12 (3.6)
No	104 (93.5)	218 (97.8)	322 (96.4)

LBW, low birth weight; NBW, normal birth weight.

Particularly in low- and middle-income nations, LBW is a serious public health concern (3). It increases the rate of newborn and infant deaths and impairment and adds considerably to the burden of childhood diseases (4). A newborn weighing less than 2.5 kg is referred to as LBW, and this is assessed immediately after birth (5). Babies born with LBW may have cerebral palsy, cognitive impairments, motor disabilities, and other psychiatric and behavioral problems (6–9).

While preterm delivery and intrauterine growth retardation are thought to be the causes of LBW, the pathophysiology of LBW is uncertain. The prevalence of LBW in Ethiopia ranges from 8% (10) to 55.9% (11). Some facility-based case–control studies have been done in Ethiopia to identify the determinants of LBW (4 12–16).

Globally, more than 20 million infants are born annually, and 15.5% are born with LBW. Of these, 95.6% are from developing countries. Compared to industrialized countries, the prevalence of LBW in poor countries is more than 20 times higher (17). Approximately 17 million infants are born with LBW in developing countries annually. Regional estimates of LBW include 28% in South Asia, 9% in Latin America, and 13% in sub-Saharan Africa (18). According to an in-depth analysis of the Ethiopian Demographic and Health Survey (EDHS) 2016, the prevalence of LBW in Ethiopia was approximately 29.1% (19). According to a study conducted in Tigray, Ethiopia, the prevalence of LBW is 6.3% in urban areas and 9.9% in rural areas (20).

Many studies have found numerous risk factors for low birth weight. In central Tigray, the sex of the neonate, fewer than four antenatal care (ANC) visits, unplanned pregnancy, and maternal food intake during pregnancy were associated with LBW (21). A study conducted at Gondar University Hospital found that low birth weight was mostly associated with pregnancy-induced hypertension, malaria during pregnancy, female newborns, and a gestational age of less than 37 weeks (22).

A number of cross-sectional and experimental investigations have found that khat consumption during pregnancy can affect pregnancy outcomes. A study of 1,141 Yemeni pregnant women found that those who chewed khat had more LBW neonates than those who did not (23). However, this study showed that there was no increase in congenital abnormalities of neonates and stillbirths born to khat-consuming mothers. Some Ethiopian studies have also shown an association between khat consumption during pregnancy and negative pregnancy outcomes (24–29). A case–control study conducted in the Bale Zone revealed a positive significant association between LBW and khat chewing. The results showed that the odds of having a low-birth-weight baby among mothers who had a history of khat chewing were six times higher than that among non-consumers (24). Similar to this, recent case–control research carried out in Jimma revealed that the likelihood of low birth weight was 12 times higher in neonates from khat-chewing mothers than in neonates from non-consumers (25).

In Ethiopia, only a few analytical epidemiological researches on the association between khat consumption during pregnancy and low birth weight have been conducted. Therefore, we conducted an unmatched case–control study that was designed to measure the association between khat consumption during pregnancy and other determinants and delivering a baby with low birth weight at Halaba Kulito General Hospital, South Ethiopia.

## Methods

### Study design and period

An institution-based unmatched case–control study was conducted in Halaba Kulito General Hospital in southern Ethiopia from 01 October 2023 to 27 February 2024. The hospital was established in 2000 and upgraded to the status of a general hospital in 2022. It is located in Halaba, one of the four districts in the Halaba Zone. The zone has five urban and 79 rural “kebeles” (the lowest administrative division in Ethiopia).

Two hospitals, four clinics, 14 health centers, and 72 health posts provide healthcare services to the population of 301,658 (150,113 men and 151,545 women). Halaba Kulito Hospital provides 24-h uninterrupted healthcare services, including specialized services for both insured and uninsured patients from diverse ethnic and socioeconomic backgrounds. It serves as one of the referral centers for the primary-level healthcare facilities in the rural and urban communities of the municipality.

### Source population and study population

The source population is all mothers who gave birth and neonates in pairs in Halaba Kulito General Hospital in Halaba Zone. The study population is all mothers who gave birth and neonates in pairs in the Halaba Kulito General Hospital during the study period.

### Eligibility criteria

#### Inclusion criteria

• A newborn weighing less than 2,500 g is referred to as LBW (5). Mothers who gave live birth to infants who weighed less than 2,500 g were considered cases, and mothers who gave live birth to infants who weighed 2,500 g and above were considered controls.
• Mothers who gave birth to single-birth infants were included.

#### Exclusion criteria for cases and controls

• Mothers who had infants with congenital anomalies were excluded from the study. In addition, mothers who were seriously ill during the data collection period were excluded. Seriously ill refers to a medical condition in which women's health is significantly compromised, often posing a risk to life or requiring intensive medical intervention.
• Those who were unable to communicate were excluded from the study.

### Sample size determination and sampling procedure

The proportion of khat use during pregnancy, khat abuse, and khat use before pregnancy were used to determine the sample size since they were the main exposure variables. Khat use during pregnancy was chosen as an independent variable since it resulted in a higher sample size for the other computed explanatory variables. The sample size was calculated using Epi Info version 7.2.5.0 with the following assumptions: 9.8% proportion of exposure (history of khat chewing during pregnancy) among control groups with an adjusted odds ratio of 2.83 (26), 95% confidence interval (*Z*_α/2_ = 1.96), 80% power (*Z*_β_ = 0.80), and a 2:1 control-to-case ratio. After adding a non-response rate of 20% (the assumption that women might not give consent after giving birth because of negative birth outcomes, pain, or exhaustion), the total sample size was 334 (111 cases and 223 controls). Eligible cases and two consecutive controls were interviewed by data collectors stationed in each health facility delivery ward until the desired sample size was reached.

### Data collection tool and procedure

Data on maternal sociodemographic and obstetric characteristics, khat use habit, and smoking and alcohol consumption were collected using a pretested questionnaire adopted from various studies (4 12–16). For maternal khat use, case and control women were asked: “How often did you chew khat during pregnancy?” The women chose one of the following responses: every day, weekly, monthly, or never. The women who chewed khat were then asked: “On the days that you chew, how many bundles of khat would you usually have? Women who chewed khat were also asked about the duration of their khat chewing sessions and their simultaneous use of khat and a water pipe.” Obstetric characteristics [gravidity, parity, ANC follow-up, iron-folic acid supplementation, pregnancy-induced hypertension, mode of delivery, and gestational age] were collected from the women's medical record card and delivery summary (Supplementary File S1).

Based on the patients’ medical record numbers, the required medical record cards were drawn from the card room. In addition to the interview and medical record review, the weight of the newborns was measured after birth. The weights of the newborns were measured within 1 h after delivery using a balanced digital Seca scale (Germany), and basic newborn care was provided. The scales were calibrated, and the reading on each scale was reset to zero prior to measuring each newborn. One trained BSc midwife working outside the respective health facilities conducted the interviews, document review, and anthropometric measurements. The data collection procedure was supervised by one MSc-certified midwife. The interviewers were blinded to the case/control status of the participants. The interview was held in a separate room after the woman was stabilized and ready to be discharged. The two respective controls and the cases were interviewed by the same interviewer.

### Data quality control

The questionnaire was first prepared in English and translated into the local language, Amharic, and then back to English by independent language experts to check its consistency. A pre-test was conducted on 17 participants out of the study setting, and necessary corrections were made accordingly. The case-to-control ratio used in the pre-test was 1:2 (6 cases and 11 controls). Four days of training were provided to the data collectors and supervisors on the objective of the study, confidentiality of information, respondents` rights, and data collection procedure prior to data collection. Weighing scales were checked and adjusted to zero to check the validity of the measurements. Continuous follow-up and supervision of data collection were conducted by the supervisors. The collected data were checked for completeness, accuracy, and clarity by the investigator and supervisors on a daily basis. Appropriate measures were taken to ensure completeness before data entry.

### Data processing and analysis

The data were checked for completeness and inconsistencies and then cleaned and analyzed in Stata (version 14.0). A descriptive analysis was carried out by calculating the mean and standard deviation for normally distributed continuous variables and proportions for categorical variables. The normality of the continuous variables was checked using the Kolmogorov–Smirnov test. Primarily, we conducted bi-variable logistic regression to select variables. The variables that were significant at a *p*-value of 0.25 in the bi-variable logistic regression analysis were candidate variables for the multivariable analysis. A multivariable binary logistic regression analysis was conducted to evaluate the association between khat chewing and adverse birth outcomes after adjusting for confounding variables. The results are presented as adjusted odds ratios (AORs) with 95% CI, which express the magnitude of the effect of each khat-related factor on the outcome relative to the reference category. Variables that were significant at a *p*-value of 0.05 and 95% CI in the multivariable logistic regression analysis were considered to be the determinant factors of delivering a baby with low birth weight. Model fitness was tested with the Hosmer–Lemeshow goodness of fit test and omnibus tests of model coefficients. Variance inflation factor and tolerance test were also used to check multicollinearity.

## Results

### Sociodemographic characteristics of participants

A total of 334 study participants (111 were cases and 223 were controls) were enrolled, resulting in a response rate of 100%. The mean age of the cases and controls was 25.4 ± 4.57 years and 24.2 ± 3.96 years, respectively. Furthermore, 58% of the cases and 30.5% of the controls had no formal education. Nearly similar proportions of the cases (68.5%) and controls (61.4%) were Muslim. Regarding place of residence, more than half (56.8%) of the cases and 13.4% of the controls were resident in rural areas. With respect to occupation status, more than two-thirds of the cases (63.1%) and controls (66.4%) were housewives (Table 1).

### Obstetric and nutritional characteristics of participants

All the mothers were non-smokers and non-water pipe smokers. The proportion of assisted emergency cesarean section (CS) was 21.6% in the cases, which was higher than the proportion in the controls (9.0%). The proportion of mothers who had four or more ANC visits among the controls was 67.3%, which was higher than that among the cases (30.6%) during the current pregnancy. A higher proportion of cases (26.1%) had a short inter-pregnancy interval than the controls (18.8%). Among the cases, 26.1% of mothers had less than a 2-year gap between the current and previous pregnancies.

Nearly similar proportions of cases (46.6%) and controls (45.0%) were multigravida, while 23.4% of the case group and 28.3% of the control group were primigravida. The majority of the women, 96.4% of the cases and 99.1% of the controls, had no history of pregnancy-induced hypertension. More than half of the cases (53.2%) and 15.2% of the controls had a mid-upper arm circumference (MUAC) of <23 cm. The majority of the participants, 90.1% of the cases and 97.3% of the controls, had no history of abortion before the preceding birth. Finally, 6% of the cases and 2.2% of the controls consumed alcohol during pregnancy (Tables 2 and 3).

### Maternal khat use and other behavioral characteristics of the participants

Concerning khat use during pregnancy, a higher proportion of case mothers used khat during pregnancy compared to control mothers (69.4% vs. 23.3%). Furthermore, 11% of the cases and 4.9% of the controls were monthly khat users, while weekly and daily khat use was recorded for 21.6% of the cases and 11.2% of the controls, and 36.0% of the cases and 7.2% of the controls, respectively. Approximately 23% of the cases and 11.0% of the controls used half a bundle of khat per session. Likewise, a higher proportion of cases (45.9%) used one bundle of khat per session than the controls (13.0%). Moreover, 41% of the cases and 13% of the controls chewed khat for 2–5 h per session. Finally, the proportion of women who chewed khat for more than 6 hours per session in the case group was higher than that in the control group (27.9% vs. 10.3%) (Table 3).

### Bi-variable analysis of low birth weight and independent variables

As shown from the results of the bi-variable analysis in Table 4, 16 variables showed a significant association with low birth weight at a 25% level of significance. During the bi-variable analysis of the sociodemographic factors, maternal age ≥26 years [COR = 2.5; (95%CI: 0.79, 8.07)], illiteracy [COR = 4.3; (95%CI: 2.22, 8.21)], and rural residence [COR = 8.4; (95%CI: 4.93, 14.45)] were found to be associated with low birth weight at a 25% level of significance. However, marital occupation and religion were not significantly associated with low birth weight.

**Table 4:** Candidate variables for multivariable binary logistic regression to identify determinants of LBW for newborns delivered in Halaba Kulito General Hospital, Halaba Zone, South Ethiopia.

Variable	LBW %	NBW %	COR (95% CI)	*p*-Value
Maternal age (year)				
≤19	3.6	7.2	1	
20–25	59.5	63.7	1.8 (0.60, 5.78)	0.284
≥26	36.9	29.1	2.5 (0.79, 8.07)	0.119
Maternal education				
Cannot read and write	58.5	30.5	4.3 (2.22, 8.21)	0.000**
Primary (1–8)	28.8	39.0	1.7 (0.84, 3.33)	0.147**
Secondary (9–12) and above	13.5	30.5	1	
Residence				
Urban	43.2	86.6	1	
Rural	56.8	13.4	8.4 (4.93, 14.45)	0.000**
Gravidity				
Primigravida	23.4	28.3	1	
Multigravida	57.6	60.5	1.1 (0.66, 1.98)	0.618
Grand multigravida	18.9	11.2	2.0 (0.97, 4.26)	0.059**
Gestational age				
<37 weeks	83.8	13.5	20.2 (11.33, 36.18)	0.000**
≥37 weeks	16.2	86.5	1	
Number of antenatal care (ANC) visits				
1 to 3 ANC visits	69.4	32.7	4.6 (2.8, 7.6)	0.000**
≥4 ANC visits	30.6	67.3	1	
Mode of delivery				
Spontaneous vaginal delivery	78.4	91.0	1	
Emergency cesarean section	21.6	9.0	2.8 (1.47, 5.33)	0.002**
Pregnancy-induced hypertension				
Yes	3.6	0.9	4.13 (0.74, 22.91)	0.105
No	96.4	99.1	1	
Maternal mid-upper arm circumference (cm)				
<23	53.2	15.2	6.3 (3.74, 10.63)	0.000**
≥23	46.8	84.8	1	
Weight gain during pregnancy				
<12 kg	92.8	79.8	3.2 (1.48, 7.17)	0.003**
≥12 kg	7.2	20.2	1	
Iron and folate supplementation				
Yes	82.0	96.9	1	
No	18.0	3.1	6.8 (2.77, 16.60)	0.000**
Have you ever chewed khat?				
Yes	69.4	23.3	7.4 (4.48, 12.39)	0.000**
No	30.6	76.7	1	
How often do you chew khat?				
Never	36.6	76.7	1	
Monthly	11.7	4.9	5.9 (2.46, 14.38)	0.000**
Weekly	21.6	11.2	4.8 (2.47, 9.44)	0.000**
Daily	36.0	7.2	12.6 (6.33, 24.98)	0.000**
Time spent in khat sessions				
0	30.6	76.7	1	
2–5 h	41.4	13.0	7.9 (4.41, 14.43)	0.000**
≥6 h	27.9	10.3	6.8 (3.53, 13.02)	0.000**
Amount of khat chewed per khat session (bundle)				
Non-user	30.6	75.8	1	
Half a bundle	23.4	11.2	5.2 (2.67, 10.01)	0.000**
One bundle	45.9	13.0	8.7 (4.86, 15.71)	0.000**
Have you ever drunk alcohol?				
Yes	6.3	2.2	2.9 (0.91, 9.47)	0.072
No	93.5	97.8	1	

** *p*-Value ≤0.25.

With regard to maternal obstetric and health-related factors, gravidity of five and more [COR = 2.0; (95%CI: 0.97, 4.26)], gestational age of <37 weeks [COR = 20.2; (95%CI: 11.33, 36.18)], between 1 and 3 ANC visits [COR = 4.6; (95%CI: 2.8, 7.6)], emergency CS ([COR = 2.8; (95%CI: 1.47, 5.33)], pregnancy hypertension [COR = 4.13; (95%CI: 0.74, 22.91)], maternal MUAC <23 [COR = 6.3; (95%CI: 3.74, 10.63)], weight gain during pregnancy <12 kg [COR = 3.2; (95%CI: 1.48, 7.17)], without iron supplementation [COR = 6.8; (95%CI: 2.77, 16.60)], maternal khat use [COR = 7.4; (95%CI: 4.48, 12.39)], monthly khat use [COR = 5.9; (95%CI: 2.46, 14.38)], weekly khat use [COR = 4.8; (95%CI: 2.47, 9.44)], daily khat use [COR = 12.6; (95%CI: 6.33, 24.98)], time spent in khat sessions 2–5 h. [COR = 7.9; (95%CI: 4.4, 14.43)], time spent in khat sessions ≥6 h [COR = 6.8; (95%CI: 3.53, 13.02)], half a khat bundle chewed per session [COR = 5.2; (95%CI: 2.67, 10.01)], one khat bundle chewed per session [COR = 8.7; (95%CI: 4.86, 15.71)], and alcohol use [COR = 2.9; (95%CI: 0.91, 9.47)] were significantly associated with low birth weight in the bi-variable analysis. Whereas parity, birth interval with previous birth, and previous history of abortion were not significantly associated with low birth weight (Table 4).

### Multivariable analysis of low birth weight and independent variables

After multivariable logistic regression analysis and controlling for possible confounders, the factors found to be significantly associated with the delivery of LBW babies were maternal education, residence, gestational age, maternal MUAC, weight gain during pregnancy, and frequency of khat use. However, maternal age, iron supplement use, mode of delivery, number of ANC visits, gravidity, and alcohol use were not significantly associated with the delivery of LBW babies. The likelihood of delivery of an LBW baby among women who had no formal education was approximately four times higher compared to those who had at least a secondary education (AOR = 3.7, 95%CI: 1.34, 10.45). Accordingly, the odds of having a low birth weight baby were four times higher among women who lived in rural areas as compared with women who lived in urban areas (AOR = 4.1, 95%CI 1.51, 11.35).

Concerning gestational age, the neonates delivered before 37 weeks of gestational age were 16 times more likely to have low birth weight than neonates delivered at ≥37 weeks of gestational age (AOR = 16.5, 95% CI 7.05, 38.55). Moreover, the odds of having a low birth weight baby among women whose MUAC was less than 23 cm were approximately five times (AOR = 4.7, 95%CI 1.89, 11.65)] higher compared to women whose MUAC was ≥23 cm. Mothers who gained <12 kg during pregnancy were at higher risk of giving birth to a low birth weight baby as compared to mothers with those who gained ≥12 kg (AOR = 4.8, 95%CI 1.22, 18.59). Regarding khat use during pregnancy, the odds of having a low birth weight baby were increased among women who used khat monthly (AOR = 9.5, 95%CI 2.13, 41.98), weekly (AOR = 11.1, 95%CI 3.69, 33.40), and daily (AOR = 14.1, 95%CI 4.74, 42.03) as compared to women who did not use it during pregnancy (Table 5).

**Table 5:** Multivariable binary logistic regression to identify determinants of LBW, Halaba Zone, Ethiopia.

Variable	LBW %	NBW %	AOR (95% CI)	*p*-Value
Maternal age (year)				
≤19	3.6	7.2	1	
20–25	59.5	63.7	1.44 (0.31, 6.78)	0.643
≥26	36.9	29.1	2.43 (0.43, 13.64)	0.314
Maternal education				
Cannot read and write	58.5	30.5	3.7 (1.34, 10.45)	0.012**
Primary (1–8)	28.8	39.0	1.1 (0.36, 3.16)	0.895
Secondary (9–12) and above	13.5	30.5	1	
Residence				
Rural	56.8	13.4	4.1 (1.51, 11.35)	0.006**
Urban	43.2	86.6	1	
Gravidity				
Primigravida	23.4	28.3	1	
Multigravida	57.6	60.5	0.4 (0.11, 1.61)	0.203
Grand multigravida	18.9	11.2	1.1 (0.42, 2.69)	0.898
Gestational age				
<37 weeks^a^	83.8	13.5	16.5 (7.05, 38.55)	0.000**
≥37 weeks	16.2	86.5	1	
Number of antenatal care (ANC) visits				
1–3 ANC visit	69.4	32.7	1.7 (0.71, 3.91)	0.239
≥4 ANC visit	30.6	67.3	1	
Mode of delivery				
Spontaneous vaginal delivery	78.4	91.0	1	
Emergency cesarean section	21.6	9.0	3.0 (0.94, 9.89)	0.063
Pregnancy-induced hypertension				
Yes	3.6	0.9	2.6 (0.14, 47.89)	0.520
No	96.4	99.1	1	
Maternal mid-upper arm circumference (cm)				
<23	53.2	15.2	4.7 (1.89, 11.65)	0.001**
≥23	46.8	84.8	1	
Weight gain during pregnancy				
<12 kg	92.8	79.8	4.8 (1.22, 18.59)	0.025**
≥12 kg	7.2	20.2	1	
Iron and folate supplementation				
Yes	82.0	96.9	1	
No	18.0	3.1	2.2 (0.40, 12.08)	0.360
How often do you have chew khat?				
Never	36.6	76.7	1	
Monthly	11.7	4.9	9.5 (2.13, 41.98)	0.003**
Weekly	21.6	11.2	11.1 (3.69, 33.40)	0.000**
Daily	36.0	7.2	14.1 (4.74, 42.03)	0.000**
Have you ever drunk alcohol?				
Yes	6.3	2.2	4.7 (0.60, 36.44)	0.139
No	93.5	97.8	1	

** *p*-Value less than 0.05.

^a^ Newborns born between 30 and 32 weeks.

## Discussion

An unmatched case–control study was conducted among women who delivered in Kulito General Hospital in southern Ethiopia to determine whether maternal khat use and other factors were associated with the delivery of an LBW baby. This study has identified some sociodemographic, obstetric, khat use-related, and lifestyle-related factors that are associated with the delivery of LBW babies in the study area.

This study found that some sociodemographic factors have a negative impact on the weight of newborns. Maternal age is regarded as an important determinant of healthy outcomes in pregnancy. This study demonstrated no statistical association between maternal age and low birth weight, which conflicts with a study conducted in Nepal that revealed a higher risk of delivering a low birth weight baby by mothers aged less than 20 and more than 30 years (30).

Furthermore, mothers who resided in rural areas were more likely to deliver LBW babies. This finding is in agreement with studies conducted in Tanzania (31) and Ghana (32). The disparity could be attributed to poor rest and constant hard labor during pregnancy among mothers in rural areas. However, this result contrasts with research conducted in the Jimma Zone, Ethiopia (33), and Bangladesh (34) where the risk of delivering low birth weight babies was found to be significantly higher in mothers who resided in urban areas than those living in rural areas. It appears that these results may be attributed to the urban women's attitude towards antenatal care, their interpregnancy gap, and iron and vitamin supplements throughout pregnancy (34).

Other research carried out in similar circumstances found that LBW was more likely among women who gained inadequate gestational weight than among women who obtained enough weight (35). Women who gained less than 12 kg during pregnancy had a three-fold higher risk of delivering an LBW baby compared to women whose weight gain was 12 kg or above. The results reported are identical to the studies conducted in Bangladesh (36) and Mozambique (37). Weight gain during pregnancy is impeded by ill health, poor sanitation, and an inadequately balanced diet, which ultimately hampers the normal growth and development of the baby.

Another factor identified in this study was the MUAC of the mother. Mothers whose MUAC measurement was below 23 cm had a higher risk of giving birth to LBW newborns than mothers whose MUAC measurement was ≥23 cm. This finding was consistent with studies conducted in Addis Ababa (38); Sidama, southern Ethiopia (39); Kersa, Oromia, Ethiopia (40); and Sawula, southern Ethiopia (41). A systematic review of 12 longitudinal studies indicated that 50% of the studies investigated the association between low maternal MUAC and LBW babies, and all of these reported a considerably elevated risk of LBW among mothers with low MUAC during pregnancy (42).

Antenatal consumption of various substances has been recognized to be significant (43). The WHO Expert Committee on Drug Dependence (ECDD) critical review results showed that substance use during pregnancy may have different obstetric effects, including low birth weight (44). Our study showed that the odds of having an LBW baby were increased among women who used khat monthly, weekly, and daily compared to women who did not use it during pregnancy. Consistent with this conclusion, studies from Africa and the Middle East show a connection between khat consumption and low birth weight (45–47). In a recent systematic review and meta-analysis, the pooled odds of giving birth to an LBW baby among mothers who used khat during pregnancy were three times higher than the non-users (48). This relationship between antenatal khat usage and LBW may be attributed to the sympathomimetic activity of cathinone, the primary element of khat responsible for its vasoconstrictive effects, which complicate pregnancy and birth outcomes. The vasoconstrictive effects include maternal tachycardia, preeclampsia, decreased placental blood flow, and fetal hypoperfusion, leading to intrauterine fetal hypoxia and limited fetal growth (49).

An experimental animal investigation found a reduction in placental blood flow due to vasoconstriction in the uteroplacental vessels among khat-fed animals compared to controls (50), which may have led to fetal growth restriction. The normal growth of the unborn fetus throughout intrauterine life is highly dependent on the healthy growth and appropriate attachment of the umbilical cord to the placenta (51). Abnormal cord insertion (marginal), abnormal umbilical cord coiling, and true umbilical cord knots were found to be significantly more prevalent among births in khat user cohorts compared to the births of their non-khat user counterparts (52).

Khat also has an anorectic effect (53–55), which could lead to decreased food intake by the pregnant woman. Chewing khat may diminish the appetite of pregnant mothers; therefore, pregnant mothers who chew khat may consume less, which may significantly decrease the nutrients for the unborn fetus and hence influence its growth. In a similar manner, an experimental study indicated that there were significant reductions in fetal weight and crown-rump length at different amounts of khat consumption (56). Furthermore, pregnant mothers who chew khat, and even those who are poor, may prioritize purchasing khat, and not have enough nutritional foods at home and thus consume less food, which may not meet the needs of the unborn fetus, affecting its growth.

### Strengths and limitations of the study

The selection of cases and controls was based on the records of the maternal and neonatal register, therefore, it is less likely that this study has misclassification biases in both the exposure and case–control categories. The most important limitation of the current study lies in the fact that the data used for the analyses were primarily collected for routine healthcare services and not for research purposes or for a specific intervention. Errors may have occurred during the documentation of the records. Another limitation of our study is that we were unable to identify whether LBW was largely caused by gestational duration or khat consumption. Future research should investigate this association by including more specific data on gestational age and other relevant variables.

Furthermore, the study analyzed data from one hospital, and the findings may not be generalizable to mothers who attended other hospitals or those who delivered at home. Another limitation of this study was that most of the information was self-reported; therefore, it was prone to reporting bias. However, we provided extensive training to our data collectors to retrieve participant's information as accurately as possible. Ideally, serum cathinone levels would have been a better measure; however, it was not possible to obtain blood samples in our study.

## Conclusion and recommendation

This study showed that sociodemographic, nutritional, obstetric, and maternal khat use factors were significantly associated with delivering an LBW baby. Rural residency, women who had no formal education, neonates delivered before 37 weeks of gestational age, women whose MUAC was less than 23 cm, and mothers who gained <12 kg were determinants of LBW. Our study underscores the importance of antenatal care and health education about the effects of khat use and other factors during pregnancy that may lead to LBW.

We recommend engagement with stakeholders in khat control to provide health education and awareness, incorporate khat use among women in khat control policy, and design interventions for khat use cessation among women. Specifically, the Ministry of Health should develop guidelines that incorporate the effect of khat on maternal health and newborn health outcomes. Additionally, health workers and local community and religious leaders should emphasize the provision of health education regarding the damage resulting from pregnant mothers chewing khat, with a special focus on the effect on their fetuses. Further studies, preferably prospective cohort studies that include evaluation of khat use at each trimester and psychological factors, are needed to identify and characterize the underlying causes of LBW in Halaba and elsewhere.

## Data Availability

The original contributions presented in the study are included in the article/Supplementary Material, and further inquiries can be directed to the corresponding author.
