# Supplementary material for: Association between maternal khat use and other determinants and low birth weight in Halaba Zone, South Ethiopia: an unmatched case–control study
Source: Front Pediatr. 2025 Feb 25;13:1416027. doi: 10.3389/fped.2025.1416027 (PMC11894810; doi:10.3389/fped.2025.1416027)
Supplement: Supplementary file 1 [file Datasheet1.docx]

**English version of data collection tool**

**Information sheet**

**Introduction:** Hello! Madam my name is ___________and I came from _________________ university. We are conducting a study on ‘Association of maternal khat use and other determinants with low birth weight in Halaba zone, South Ethiopia: Unmatched case-control study’. The aim of this study is to identify maternal khat use, maternal socio-demographic and obstetric risk factors associated with low birth weight in the Halaba Kulito zone, South Ethiopia. The results of this study will be helpful to improve the health of newborn, and provide evidence for policymakers to fully understand potential determinants that affect birth weight. Recommendations will be made to responsible organizations based on the findings.

**Benefits:** Participation in this study may provide no direct benefit to the facility or participants. However, the information we will obtain is expected to assist service providers and policymakers working in newborn health in learning about the health effects of khat and other determinants on newborns, which will be useful in developing strategies to improve the health of newborns.

**Possible Risks:** Except for a few minutes spent answering questions, there was no risk associated with participation in this study.

**Confidentiality:** The names of women will not be included in the data collection form in order to keep the information obtained confidential. The data will only be used for research purposes. The confidentiality of the responses will be guaranteed. All data will be stored on pass word-protection devices and will be deleted from all computers use for data entry once uploaded to the secured study laptop. All appropriate measures will be taken to protect women’s privacy during data collection.

**Consent statements (to be read by data collector for the study participants)**

I am going to ask you questions. You are randomly selected to participate in the study. Your participation in this study doesn’t involve any direct risk or benefit for you, but is very useful to improve the health of newborns in this area. Your name will not be appearing on this questionnaire, and all the information you provide me will be strictly confidential. It takes 30 minutes to finish the interview and you are not obliged to answer any question you don’t wish to answer, and you can also end this interview at any time, if you wish to do so.

Would you like to participate in the study?

Yes________ No_________

Contact address of principal investigator: - mobile: +251912620885, +251916270201

Email: [biruk900zelalem@gmail.com](mailto:biruk900zelalem@gmail.com)

Participant code number: _____________________________

Date (DD/MM/YY): ______________________ Time (local time) ____________

Participant card number: _________________________________________

| **Part 1: Socio-demographic characteristics and coffee consumption** | | | |
| --- | --- | --- | --- |
| 100 | How old are you? | ( _______ ) years | |
| 101 | What is your highest educational level? | 1.No education 2.Complete grade (Grade 1-8 )  3. Complete grade (Grade 9-12) 4. More than secondary (Above grade12) | |
| 102 | What is your current occupation? | 1. Housewife 2. Merchant 3.Employed 4.Other (Please specify:____________ ) | |
| 103 | What is your religion? | 1.Muslim 2.Orthodox 3.Protestant 4.others (specify _________ ) | |
| 104 | Where do you live? | 1. Urban 2.Rural | |
| **Part 2: Pregnancy and obstetric characteristics pregnant women** | | | |
| 200 | How many pregnancies do you have? **(To be filled from mother cards)** | | ( _______ ) pregnancies |
| 201 | How many deliveries have you had? **(To be filled from mother cards )** | | ( _______ ) deliveries |
| 202 | What is the duration between the current & previous childbirth in years? | | 1. ≤2 years 2. >2 years |
| 203 | How many times do you have visited the ANC clinic for the current pregnancy? **(To be filled from mother cards )** | | ( _______ ) visits |
| 204 | Mode of delivery **(To be filled from mother cards )** | | 1. Spontaneous vaginal delivery 2.Caesarian section 3. Instrumental |
| 205 | Have you had history of abortion? **(To be filled from mother cards)** | | 1. Yes 2. No |

| **Part 3: Medical history related characteristics of mothers** | | | | |
| --- | --- | --- | --- | --- |
| 300 | Have you developed hypertensive disorders during the current pregnancy? **(To be filled from mother cards)** | | | 1. Yes 2. No |
| **Part 4: Nutritional, khat use and alcohol drinking related characteristics of mother** | | | | |
| 400 | Maternal MUAC measurement in centimeters **(To be filled from mother cards)** | ______________ | | |
| 401 | Did you get iron folic acid supplementation during this current pregnancy? (**To be filled from mother cards)** | 1.Yes 2.No | | |
| 402 | What is your weight before pregnancy and during pregnancy (in kg)? | ______________ | | |
| 403 | Do you chew khat during pregnancy ? | 1.Yes 2.No | | |
| 404 | How often do you have chew khat during pregnancy? | 1.Never 2.Monthly 3.Weekly  4. Daily | | |
| 405 | Time spent in khat sessions(hours) during pregnancy | __________ | | |
| 406 | Amount of khat chewed per khat session(bundle) during pregnancy | ___________ | | |
| 407 | Do you drink alcohol during pregnancy? | 1.Yes 2.No | | |
| 408 | Do you smoke cigarette during pregnancy? | 1.Yes 2.No | | |
| 409 | Do you smoke water-pipe during khat chewing? | 1.Yes 2.No | | |
| **Part 5: Birth outcome assessment (To be filled from mother cards & measurement)** | | | | |
| 500 | Preterm birth (Before 37 completed weeks of gestation)? | | 1. Yes 2. No | |
| 501 | Low birth weight (Birth weight below 2500 g)? | | 1. Yes 2. No | |
| 502 | Still birth (Infant delivered with no sign of life)? | | 1. Yes 2.No | |
| 503 | Birth defect/congenital abnormality (structural changes in one or more parts of the body)? | | 1. Yes 2. No | |
| 504 | Gestational age | | 1. Yes 2. No | |

**Amharic version data collection tool**

የተሳታፊ ኮድ ቁጥር፡- _________________________

ቀን (ቀን/ወወ/ዓ.ዓ)፡ ___________________________

የተሳታፊ ካርድ ቁጥር፡ ________________________

| **ክፍል 1፡ ማህባረዊና ኢኮኖሜያዊ ሁኔታዎችን የሚደስስ መጠይቅ** | | | | | | | |
| --- | --- | --- | --- | --- | --- | --- | --- |
| 100 | | | እድሜዎ ስንት ነው? | (______) ዓመታት | | | |
| 101 | | | ከፍተኛ የትምህርት ደረጃዎ ምንድ ነው? (በትምህርት የዓመታት ብዛት) | 1.አልተማርኩም 2.1-8ኛ ክፍል  3. ከ9-12ኛ ክፍል) 4. ከሁለተኛ ደረጃ በላይ (ከ12ኛ ክፍል በላይ) | | | |
| 102 | | | አሁን ያለህበት ሙያ ምንድን ነው? | 1. የቤት እመቤት 2. ነጋዴ 3.ተቀጣሪ 4.ሌላ (እባክዎ ይግለጹ፡____________) | | | |
| 103 | | | ሃይማኖቶ ምንድን ነው? | 1.ሙስሊም 2.ኦርቶዶክስ 3.ፕሮቴስታንት 4.ሌሎች (________ ይግለጹ) | | | |
| 104 | | | የት ነዉ የሚኖሩት? | 1. ከተማ 2. ገጠር | | | |
| ክፍል **2፡** ከእርግዝና ጋር ተያያዝነት ያላቸዉ ጥያቄዎች | | | | | | | |
| 200 | ስንትኛ እርግዝናዎ ነዉ ? | | | | (_______) | | |
| 201 | ስንት ልጆች አሎት ? | | | | (_______) | | |
| 202 | ለአሁኑ እርግዝና ምን ያህል ጊዜ ጤና ተቁዓም ላይ ክትትል አድርገዋል? | | | | (_______) | | |
| 203 | ምን ያህል ግዜ ቆይተዉ ነዉ ያረገዙት ከቀድሞ እርግዝና | | | | 1. <2 2. ≥2 አመት | | |
| 204 | ቀደም ሲል ዉርጃ አጋጥመዋት ያዉቃል ? | | | | 1.አዎ 2. የለም | | |
| 205 | እንዴት ነዉ የወለዱት | | | | 1.በሰርጀሪ 2.ያለ ሰርጀሪ | | |
| 3.የጤና እክልን በተመለከተ | | | | | | | |
| 300 | እርግዝና ወቅት የደም ግፊት በሽታ ታሪክ ነበረዎት? | | | | | | 1.አዎ 2. የለም |
| 4. አመጋገቦን፤ የጫትና የአልኮል አጠቃቀም በተመለከተ | | | | | | | |
| 400 | | በእርግዝና ወቅት አይርን ፎሊክ አሲድ ወስደዋል? | | | | 1.አዎ 2. የለም | |
| 401 | | ከማርገዞ በፊት እና በእርግዝና ወቅት ክብደቶ ስንት ነዉ ? | | | | ---------- ፤----------- | |
| 402 | | ሙአክ በሴንቲሜትር | | | | ----------- | |
| 403 | | ጫት ቅመሽ ታዉቂያለሽ በእርግዝና ወቅት | | | |  | |
| 404 | | ምን ያህል ግዜ ቅመዋል በእርግዝና ወቅት | | | | 1.በፍፁም 2.በወር 3.በሳምንት 4.በየቀኑ | |
| 405 | | ለምን ያህል ስዓት ይቅማሉ በእርግዝና ወቅት | | | | ----------- | |
| 406 | | ምን ያህል ጫት ይቅማሉ በእርግዝና ወቅት | | | | ----------- | |
| 407 | | አልኮል ጠጥተዉ በእርግዝና ወቅት ታዉቂያለሽ | | | | 1.አዎ 2. የለም | |
| 408 | | ሲጋራ አጭሰዉ ታዉቂያለሽ በእርግዝና ወቅት | | | | 1.አዎ 2. የለም | |
| 409 | | ሺሻ አጭሰዉ ያዉቃሉ በእርግዝና ወቅት | | | | 1.አዎ 2. የለም | |
| 5. የወሊድ ውጤት | | | | | | | |
| 500 | | ቅድመ ወሊድ (ከ 37 ሳምንታት እርግዝና በፊት)? | | | | | 1.አዎ 2. የለም |
| 501 | | ዝቅተኛ የልጅ ክብደት (የልደት ክብደት ከ 2500 ግ በታች)? | | | | | 1.አዎ 2. የለም |
| 502 | | ሞት? | | | | | 1.አዎ 2. የለም |
| 503 | | አከላዊ ጉድለት? | | | | | 1.አዎ 2. የለም |
| 504 | | የእርግዝና ዕድሜ | | | | | ------------ |
